# Tyrosine Kinase Inhibitors for Radioactive Iodine Refractory Differentiated Thyroid Cancer

**DOI:** 10.3390/life14010022

**Published:** 2023-12-22

**Authors:** Christos Cortas, Haris Charalambous

**Affiliations:** Medical Oncology Department, Bank of Cyprus Oncology Centre, Nicosia 2006, Cyprus; christos.cortas@bococ.org.cy

**Keywords:** thyroid cancer, differentiated thyroid cancer, radioactive iodine refractory, tyrosine kinase inhibitors

## Abstract

Patients with differentiated thyroid cancer usually present with early-stage disease and undergo surgery followed by adjuvant radioactive iodine ablation, resulting in excellent clinical outcomes and prognosis. However, a minority of patients relapse with metastatic disease, and eventually develop radioactive iodine refractory disease (RAIR). In the past there were limited and ineffective options for systemic therapy for RAIR, but over the last ten to fifteen years the emergence of tyrosine kinase inhibitors (TKIs) has provided important new avenues of treatment for these patients, that are the focus of this review. Currently, Lenvatinib and Sorafenib, multitargeted TKIs, represent the standard first-line systemic treatment options for RAIR thyroid carcinoma, while Cabozantinib is the standard second-line treatment option. Furthermore, targeted therapies for patients with specific targetable molecular abnormalities include Latrectinib or Entrectinib for patients with NTRK gene fusions and Selpercatinib or Pralsetinib for patients with RET gene fusions. Dabrafenib plus Trametinib currently only have tumor agnostic approval in the USA for patients with BRAF V600E mutations, including thyroid cancer. Redifferentiation therapy is an area of active research, with promising initial results, while immunotherapy studies with checkpoint inhibitors in combination with tyrosine kinase inhibitors are underway.

## 1. Introduction

Thyroid cancer (TC) is the most common malignancy of the endocrine system, and it is estimated that approximately 585,000 new cases were diagnosed globally in 2020, which is about 3% of all cancer cases [1]. Around 75% of patients that are diagnosed with TC are women, with an incidence rate of 10.1/100,000 females per year whereas the incidence rate for males is 3.1/100,000, annually. Over the last three decades there has been a worldwide increase in the incidence of thyroid cancer which may be partly the result of overdiagnosis due to increased use of radiological investigations, including neck ultrasound and cross-sectional imaging; however there is also evidence of an increase in larger clinically significant tumors [2].

TC is divided according to the cell of origin, if it originates from follicular epithelial cells or parafollicular C cells. Parafollicular C cells give rise to medullary thyroid cancer, whereas follicular cells give rise to four histological types: papillary thyroid cancer (PTC), which accounts for 80–85% of TC, follicular thyroid cancer (FTC) accounts for 10–15%, poorly-differentiated thyroid cancer (PDTC) accounts for 1–2%, and anaplastic thyroid cancer (ATC), which accounts for less than <2%. Well-differentiated thyroid cancers (DTC) include PTC and FTC, as well as subtypes like follicular–oncocytic thyroid carcinomas (FTC-OV) and Hurthle cell carcinoma. As DTC accounts for most TCs, in this review we are going to discuss systemic treatment options for DTC [3].

DTC is mainly diagnosed at an early stage and is treated with surgery with or without adjuvant radioactive iodine (RAI) treatment and TSH suppression. DTC is one of the most curable cancers, with excellent long-term prognosis, but up to 20% of patients develop local or regional recurrences, and up to 10% develop distant metastases [4]. With longer follow ups, up to 35% of patients with DTC can recur after 40 years, with 20% failing to concentrate iodine, resulting in overall close to 10% of patients dying of their disease [5,6,7]. A study looking at the SEER database showed that independent predictive factors for distant metastasis are tumor size, age at diagnosis, thyroidectomy, node positive disease, T3-T4 stage, and histopathology for female DTC patients [8].

It is worth pointing out that even patients with metastatic disease have a much better prognosis compared with other solid tumors, with the 10-year survival being about 45% due to the use of RAI for metastatic disease. A little more than half of patients with metastatic disease, around 60%, will eventually develop disease refractory to RAI, because cancer cells lose their ability to concentrate iodine. Resistance to RAI is a poor prognostic factor and once patients develop RAIR TC, the five-year overall survival is quoted to decrease down to 10% [9].

In the past there were limited and ineffective options for systemic therapy for RAIR, but over the last ten to fifteen years the emergence of tyrosine kinase inhibitors (TKIs) has provided important new avenues of treatment for these patients, and these inhibitors are the focus of this review. Currently, Lenvatinib and Sorafenib, multitargeted TKIs, represent the standard first-line systemic treatment options for RAIR thyroid carcinoma, while Cabozantinib is the standard second-line treatment option.

In this review, we aim to present up to date information regarding the molecular biology of DTC, the role of RAI, and the definition of RAIR DTC and discuss timing and options for systemic therapy, with an emphasis on treatment with tyrosine kinases inhibitors for patients with RAIR disease, including associated toxicity and management of this toxicity. Finally, we discuss redifferentiation therapy, targeted therapies, and evidence regarding immunotherapy, as well as ongoing studies to provide an overview of the systemic management of RAIR DTC.

## 2. Methods

A PubMed search was conducted by searching the terms “thyroid cancer”, “refractory”, and “kinase inhibitor” ((“thyroid cancer”[Title/Abstract]) AND (“refractory”[Title/Abstract])) AND (“kinase inhibitor”[Title/Abstract]) in order to review current literature for the period 2007 to 2023. A total of 165 articles were identified, from which 26 articles were presenting data from clinical trials for DTC. All 26 articles were reviewed. 

Additionally, a search via clinicaltrials.gov was conducted to document all trials of the treatment of DTC with the use of tyrosine kinase inhibitors. Conditions disease was determined as thyroid cancer and tyrosine kinase inhibitors was entered in “other terms”. Clinical trials referring to medullary or anaplastic cancer were excluded. All recorded trials were reviewed to see whether they address the relevant article issue and for the presence of trial results. The trials that were not found via a PubMed search but had results in clinicaltrials.gov were individually searched via the PubMed search engine.

Trials examining redifferentiation of RAIR DTC were searched via the PubMed search engine and in clinicaltrials.gov using the same method. Finally, recent review articles were also examined for the presence of other trial or treatment options.

## 3. Molecular Biology of Differentiated Thyroid Cancer

In the pathogenesis of DTC, somatic mutations are involved affecting the RAS (Rat Sarcoma Virus)/BRAF (B-Raf proto-oncogene, Serine/Threonine Kinase), MAPK (Mitogen-Activated Protein Kinase) pathway, and PI3K (phosphatidylinositol 3-kinase)/Akt (Protein Kinase B) pathways. In PTC, beyond mutations in BRAF and RAS, fusions of the RET (Rearranged during Transfection) and NTRK (Neurotrophic Tyrosine Receptor Kinase) transmembrane proteins are also seen. In FTC, the most common events are mutations to RAS and PAX8/PPARy (Paired Box 8/Peroxisome Proliferator-Activated Receptors) rearrangements. During cancer development, proliferation and dedifferentiation, additional mutations that affect the PI3K-AKT pathway, are also generated [10].

Data from the Cancer Genome Atlas (TCGA) project, published in 2014, identified a large number of tumor driver mutations [11]. In fact, in only 3.5% of PTC cases there were no putative pathologic mutations identified. Known driver mutations identified include RAS, RET/PTC, TP53, TRK, PTEN, β-catenin, PAX8/PPARy, BRAF, PIK3CA, BRAF/AKAP9, AKT1, ETV6/NTRK3, and STRN/ALK [12]. According to the TCGA, driver mutations are mostly point mutations accounting for 75% of all cases, while approximately 15% are gene fusions and the rest are gene amplifications.

The TCGA provides evidence that BRAF alterations are the most common causative molecular event in PTCs, and it is found in approximately 58.5% of PTC cases [11]. BRAF V600E is the most common mutation found in the BRAF gene. BRAF mutations are also associated with worse prognosis in terms of overall survival, need of re-operation, and higher risk of local recurrence in patients older than 65 years old [13,14,15]. Also, BRAF mutations are thought to lead to loss of the ability of follicular cells to concentrate iodine uptake, hence leading to dedifferentiated RAIR disease [15,16,17,18]. Finally, it should be borne in mind that BRAF mutations on nodules with inconclusive cytology results from FNA are rarely found in benign neoplasms, since 99% of nodules with BRAF mutations are positive for malignancy on final histopathology [19].

The second most common genetic alteration in DTC are point mutations in RAS family genes (KRAS, NRAS, and HRAS). NRAS mutations are the most common in TC and they are predominantly alterations in exons 12 and 61 [20]. Mutations in RAS genes are identified as driver mutations in approximately 12.7% of cases according to TCGA. They are found especially with follicular histology, and sometimes in patients with PTC too [11]. Most of the cases of RAS-mutated FTCs are follicular variant PTCs. RAS mutation is also found in follicular adenomas of the thyroid, which have a high risk of progressing to malignancy. This suggests that RAS mutations are an early event in the carcinogenesis pathways, and further mutational events are needed for cancer development [20].

RET/PTC rearrangements are accounted for as the causative genetic alteration in 6.3% of PTC, according to TCGA [10]. RET is a transmembrane protein with tyrosine kinase activity to its intracellular domain which regulates both RAS and PI3K-AKT pathways. During RET/PTC rearrangement the protein product remains in a constant activated form, leading to enhanced cell proliferation. They are the result of chromosomal rearrangements and the most common are paracentric chromosomal inversions leading to RET/PTC1 and RET/PTC3 [21]. The prevalence of these mutations is higher in younger patients [22]. RET/PTC is directly correlated with exposure to ionizing radiation, based on data gathered after the Chernobyl nuclear factory accident [23]. RET/PTC rearrangements are more common in patients younger than 45 years old [24].

Another genomic alteration in PTC is the amplification of PIK3CA, present in 53.1% of patients [25]. PIK3CA amplification leads to enhanced signaling of PI3K, which has a fundamental role in thyroid cancer carcinogenesis. PIK3CA is normally activated by tyrosine kinase proteins or by RAS signaling [26]. There are also mutations in PIK3CA in 2% of patients diagnosed with PTC. They are more frequent in FTC, poorly differentiated, anaplastic, and undifferentiated TCs, reaching 10–15% [25,27].

Telomerase reverse transcriptase (TERT) mutations and TP53 mutations were also identified in patients with DTC. TERT mutations lead to amplified telomerase activity and enhanced proliferative potential of cancer cells. TERT mutations are found more frequently in poorly differentiated TC and only account for 1% of DTC, however they are associated with poor prognosis and higher risk of relapse [12]. A synergistic effect as a poor prognostic factor was observed between TERT, RAS, and BRAF mutations, leading to tumors with more aggressive behaviors [28].

TRK fusions are found in 2% of adult patients diagnosed with PTC but are higher in pediatric and young adult PTCs, where they account for about 6–15% of all PTCs [29,30,31]. TRK is normally expressed in embryogenesis and in adult life it takes part in neurological functions such as pain, proprioception, appetite, and memory. TRK fusions lead to overexpression of the chimeric protein, resulting in constitutively active, ligand-independent downstream signaling which promotes carcinogenesis.

ALK fusions are found in about 0.8% of PTCs. Case reports of RAIR thyroid cancer treated with Crizotinib are described in the literature. The most typical histologic appearance of ALK fusion-positive thyroid cancer is PTC, however, it is found with increased relative incidence in up to 4–9% of patients with PDTC [32].

Finally, PAX8/PPARy rearrangements are the result of a fusion of PAX8, which is a transcription factor, with the PPARy gene. This mutation is mainly found in FTC and in a follicular variant of PTC. PAX8/PPARy rearrangements have an incident rate of 30–35% in patients with FTC [33].

From the above, it becomes apparent that undertaking molecular testing with next-generation sequencing (NGS) for all patients developing RAIR is important, as it may facilitate targeted therapies and clinical trial participation, while the presence of some mutations may have prognostic importance [34]. Table 1 presents the prevalence of genetic alterations according to different types of TCs.

## 4. Radioactive Iodine Treatment (RAI) and Radioactive Iodine Refractory (RAIR) Disease

The hallmark of treatment of DTC in patients with localized and locally advanced disease remains surgery followed by RAI treatment and TSH suppression. RAI is part of the adjuvant treatment for patients with high-risk features. In the adjuvant setting, RAI ablates both residual cancer cells to reduce the risk of cancer recurrence and also ablates remnant thyroid tissue to facilitate follow up with thyroglobulin monitoring [36]. Post-RAI treatment whole body iodine imaging should be assessed in all cases of RAI treatment to evaluate efficacy and evidence of residual thyroid cancer.

RAI is also the standard first-line treatment for recurrent or metastatic disease. The treatment outcome and response to RAI treatment should be assessed with an iodine whole body scan. However, in up to 15% of patients, RAI treatment is no longer effective as a result of loss of the expression of the sodium iodide symporter (NIS), which occurs following loss of thyroid differentiation [37]. This results in no RAI uptake in the post-RAI scan. In patients with iodine-negative post-treatment scans, but with strong clinical or radiological suspicion of recurrence of metastatic disease, there is the option of undertaking an FDG PET/CT scan, because FDG PET/CT scans can identify lesions from tissues that are non-iodine avid [38]. In these patients, the disease is classified as RAI Refractory (RAIR) disease.

There is no global agreement regarding the definition of RAIR. It has been proposed that RAIR can occur in any of the following four conditions: (a) the absence of uptake of RAI in all lesions on scintigraphy, (b) the absence of RAI uptake in some but not all lesions, (c) progression despite uptake of RAI, or (d) reaching the maximum recommended activity of RAI [39].

On the cellular level, iodine is used by follicular cells to synthesize thyroid hormones and enters the cell by passing through the basal membrane, making use of the sodium iodine symporter mechanism (NIS). Once iodine is inside the thyroid follicle, it gets oxidized by TPO (thyroid peroxidase) and then is further processed by TG (thyroglobulin) before becoming T3 and T4 hormones. The whole process is regulated by the thyroid stimulating hormone (TSH) which binds on the TSH receptor (TSH-R). TSH-R activates adenylyl cyclase, resulting in the accumulation of cyclic AMP (cAMP) within thyroid cells. cAMP induces NIS transcription by stimulating thyroid-specific transcriptional factors (TTFs) including paired box 8 (PAX8). Activation of both MAPK/ERK and PI3K/AKT pathways results in inhibition of TTFs and loss of NIS expression [37]. The BRAF V600E mutation has been correlated to the loss of NIS expression and RAIR [40]. BRAF activation represses PAX8 binding to the NIS promoter, and results in dysregulation of involved proteins in this process e.g., NIS, TPO, TG, TSH-R, and thus results in the emergence of RAIR TC.

## 5. Localized Treatment and Timing of Initiation of Systemic Therapy for RAIR DTC

Not all patients with a rising thyroglobulin and a negative diagnostic iodine scan have RAIR metastatic disease. These patients require further investigations, including conventional neck, brain, thorax, abdomen, and bone imaging, while, increasingly, PET scans are being used to detect recurrent or metastatic disease. Causes of a false negative iodine scan also need to be excluded; the TSH level at the time of the diagnostic scan must be elevated to or above 30 mU/L, and iodine contamination (e.g., history of recent iodine contrast radiography) or a high iodine diet also need to be excluded [41]. Finally, some centers, to be absolutely sure that this is iodine refractory disease, advocate the use of high-dose iodine therapy in patients with raised thyroglobulin and a negative diagnostic iodine scan [41].

Once RAIR has been confirmed, all patients with recurrent RAIR disease, and in the absence of specific contraindications, should have TSH suppression aimed at a serum level of <0.1 μIU/mL [42]. In a small minority of patients with RAIR and isolated recurrence, confirmed on PET to be in the neck or with single or very limited oligometastatic disease, an attempt to offer surgery to render the patient disease-free may be indicated and is normally reserved for patients with excellent performance status and lack of significant comorbidity [42,43].

For patients with more extensive disease or patients with oligometastatic disease who cannot have surgery, other local modality therapies, e.g., radiotherapy or ablative techniques, may be considered with the aim to obtain disease control or palliate disease-related symptoms. Radiofrequency ablation (RFA) can be considered for solitary lesions, or for lesions causing symptoms and for lesions <2–3 cm in patients not eligible for surgery or requiring extensive resection [43]. Vertebroplasty can also be considered for patients with vertebral metastases. Bisphosphonates or denosumab should be considered in patients with TC and multiple bone metastases [43]. These decisions should be taken jointly at the Multi-Disciplinary Team (MDT) meeting, after discussion with surgical, medical, and radiation oncologists, alongside nuclear medicine physicians and endocrinologists.

For patients with multiple foci of metastatic disease, e.g., multiple lung metastases or multiple bone metastases, the question then becomes as to the timing of the initiation of the systemic therapy. An increase in serum thyroglobulin (Tg) levels in the absence of radiologically evident disease progression should not be used to select patients requiring systemic therapy [43]. Instead, the rate of Tg rise or Tg doubling time should be used to guide frequency of imaging during follow up, in conjunction with tempo of disease as judged by previous imaging. For patients with Tg antibodies, these commonly result in false negative Tg results, but they can also produce false positive Tg results [41], thus, physicians should depend on radiological imaging for monitoring disease activity/tempo of the disease and not make decisions regarding follow up of patients based on the Tg result.

Care should be exercised regarding the timing of the initiation of systemic therapy for patients with RAIR, as current treatments are non-curative, they are aimed at palliation of symptoms and prolongation of survival; these treatments are essentially life-long and are associated with significant toxicity [34,42,43]. Given that many patients may be asymptomatic and with a slow disease tempo, many patients may not need to start systemic therapy immediately, and instead a period of observation would be indicated. This period of observation in some patients may extend for years, as the natural history of DTC is quite variable, with rates of disease progression ranging from a few months to many years. However, for patients with heavy disease burden, more rapid disease tempo, and who are symptomatic from their disease, initiation of systemic therapy with tyrosine kinase inhibitors is appropriate [42,43].

## 6. Chemotherapy for RAIR DTC

For decades, conventional cytotoxic systemic therapies have been in use for patients with RAIR, however, with minimal efficacy. Doxorubicin was the most often used chemotherapy drug but with limited success; the French group of Droz and Schlumberger in 1990 reported a study involving 49 patients with non-anaplastic TC treated with doxorubicin or doxorubicin combinations with only a 3% response rate [44]. Studies of combination chemotherapy did not show significant advantage over single-agent doxorubicin, and life-threatening complications occurred more often in patients treated with the combination chemotherapy [45].

Most of the studies were undertaken in the 1970s, 1980s, and 1990s prior to the introduction of the RECIST criteria in 2000. More recent studies have shown similarly poor results. For instance, a study by Matuszczyk et al. in 2008 with 22 patients with RAIR treated with doxorubicin between 2000 and 2005 showed a 5% RR and 42% SD [46]. Equally, another study by Argiris et al. in 2008 reported on the combination of doxorubicin and interferon alpha in 15 patients with progressive RAIR DTC, and 2 with anaplastic thyroid carcinoma. In 16 patients assessable for response, 1 patient (6%), who had follicular carcinoma achieved a PR response and 10 patients (62.5%) had SD disease as best response. The median time to progression was 5.9 months and median overall survival was 26.4 months. However, there was significant toxicity, with grade 3/4 neutropenia in 76% of patients and neutropenic fever in 24%, while other grade 3/4 toxicity included fatigue (41%), rigors (18%), fever (6%), nausea/vomiting (29%), anorexia (29%), stomatitis (24%), vision disturbances (18%), neuropathy (18%), and hyponatremia (6%) [47].

With the introduction of tyrosine kinase inhibitors (TKIs), there is no further use of chemotherapy for patients with RAIR.

## 7. Multikinase Tyrosine Kinase Inhibitors and Treatment of RAIR DTC

The development of multikinase tyrosine kinase inhibitors (TKIs) with effects on multiple tyrosine kinases and also acting on angiogenesis by inhibiting Vascular Endothelial Growth Factor Receptor (VEGFR) provided new effective drugs with the potential to improve outcomes in terms of disease and symptom control, survival, and improved quality of life. All phase III randomized clinical trials referring to multitargeted TKIs for RAIR DTC are presented in Table 2.

The first TKI which was approved for the treatment of RAIR DTC was sorafenib in 2013. Sorafenib is a small molecule that acts as an inhibitor of VEGFR 1–3, PDGFR, RET, FLT, and c-kit. The drug approval was based on the DECISION trial. DECISION was a phase 3, double blind, multicentered, placebo-controlled trial, which examined the efficacy and safety of sorafenib in patients with metastatic or locally advanced RAIR TC. The primary endpoint was determined as progression-free survival (PFS), whereas secondary end points were overall survival (OS), time to progression, objective response rate (ORR), and duration of response (DoR). In the intention to treat (ITT) population (*n* = 417), sorafenib achieved a statistically significant improvement in terms of PFS against placebo (10.8 months vs. 5.8 months, HR 0.59, *p* < 0.0001). ORR on the sorafenib arm was 12.2%. OS was not statistically different between two arms, but it should be borne in mind that crossover between the two groups was permitted in the event of progression in the placebo group. Importantly, drug interruption, reduction, and withdrawal occurred in 66.2%, 64.3%, and 18.8% of the patients, respectively, with the most common cause for this being hand and foot syndrome (HFS). Other common adverse effects were diarrhea, rash, fatigue, weight loss, and hypertension [48].

Lenvatinib is a multitarget TKI that inhibits VEGFR 1-4, PDGFR, FGFR, RET, and c-kit. It gained approval for the treatment of RAIR TC based on the SELECT clinical trial. SELECT was a phase 3, randomized, double-blind, multicenter study with patients with RAIR DTC examining the efficacy of Lenvatinib 24 mg daily over placebo. The primary endpoint was PFS, while secondary endpoints were OS and ORR. Lenvatinib extended PFS versus placebo to 18.3 months compared to 3.6 months for placebo (HR 0.21, *p* < 0.0001). The ORR to Lenvatinib was 64.8% [49]. There was no statistically significant difference between the two groups in terms of OS. This was probably the result of crossover, like in the DECISION trial, given that patients in the placebo arm were able to receive Lenvatinib after disease progression. However, in a post hoc analysis of the SELECT trial, median OS was significantly longer in the Lenvatinib arm compared to the placebo arm in patients over the age of 65 years [55]; this may suggest that treatment delay in the elderly may affect survival more than in younger patients. Equally, it can be argued that disease in the elderly was more aggressive given the different median OS in the placebo groups between younger and older patients, which was not reached in patients aged ≤65 years versus 18.4 months in adults aged >65 years [45]. Finally, the Lenvatinib group had a high rate of adverse effects, with grade 3–5 adverse effects affecting 75.9% of the patients. Treatment-related toxicity included hypertension, proteinuria, renal failure, hepatic failure, and venous and arterial thromboembolic events [56]. Similar results were seen in a randomized study of Lenvatinib versus placebo from China [50].

While Lenvatinib is currently being used widely as a first-line treatment for patients diagnosed with RAIR DTC, there are concerns regarding its toxicity according to its SPC (Summary of Product Characteristics) [57], especially for older patients (>75 years), patients of Asian race, with comorbidities (such as hypertension, and hepatic or renal impairment), or body weight less than 60 kg, who appear to tolerate the drug poorly. In view of this toxicity, a trial was enacted to examine whether a lower initial dose of Lenvatinib would have similar efficacy and lower toxicity than the standard 24 mg. In a multicenter, double blind, non-inferiority, phase 2 study, Lenvatinib was examined on the standard 24 mg monotherapy dose, versus an experimental lower dose of 18 mg. In this study, dose reduction was permitted according to standard clinical practice in case of treatment-related toxicity, applying masking procedures. The results of the study were that the 18 mg starting dose failed to show non inferiority versus the standard dose, and ORR at wk24 was 57.3% in the Lenvatinib 24 mg arm and 40.3% in the Lenvatinib 18-mg arm, failing to meet the predefined odds ratio non-inferiority margin. More crucially, there was no statistically significant difference between the incidence of grade ≥ 3 treatment-related adverse events, which were 61.3% in the Lenvatinib 24 mg arm and 57.1% in the Lenvatinib 18 mg arm: a difference of −4.2%. Hence, the conclusion from this study was that the approved starting dose of Lenvatinib 24 mg/day should be continued to be used and clinicians can subsequently adjust the dose as necessary [58].

Interestingly, in a retrospective Japanese study, the toxicity and efficacy of Lenvatinib in patients having planned treatment holidays was improved, with both a PFS and OS survival benefit. This would suggest that treatment breaks, as, for instance, is practiced with sunitinib in patients with metastatic kidney cancer, may be an option to consider in patients suffering toxicity during treatment with Lenvatinib. This strategy, however, should not be used in patients tolerating this drug well [59].

COSMIC-311 was a global, randomized, double-blind, phase 3 trial of Cabozantinib 60 mg as second- or third-line treatment versus placebo in patients with RAIR DTC. Patients must have received previous Lenvatinib or sorafenib and progressed during or after treatment with up to two VEGFR tyrosine kinase inhibitors. Patients receiving placebo could cross over to open-label Cabozantinib upon disease progression. In the interim analysis, the primary endpoint of PFS was met in the ITT population. Cabozantinib displayed significant improvement in PFS over placebo: median not reached (CI 5.7–not estimable) versus 1.9 months (1.8–3.6 months); (hazard ratio 0.22; *p* < 0.0001). ORR in the Cabozantinib group was 15% versus 0% in the placebo group (*p* = 0.028). Grade 3 or 4 adverse events occurred in 57% of the 125 patients receiving Cabozantinib and 26% of the 62 patients receiving placebo. The most frequent were palmar–plantar erythrodysesthesia (10% vs. 0), hypertension (9% vs. 3%), and fatigue (8% vs. 0). Serious treatment-related adverse events occurred in 16% patients in the Cabozantinib group and 2% in the placebo group [51].

REALITY was a Chinese multicenter, double-blind, phase 3 trial of Apatinib, a VEGFR-2 inhibitor, which was compared against placebo in patients with progressive or locally advanced RAIR DTC. Apatinib proved to be superior compared to placebo in terms of median PFS, which was the primary endpoint (22.2 months vs. 4.5 months, HR 0.26, *p* < 0.0001) [57]. Furthermore, there was an improvement in confirmed ORR, which was 54.3%, and the DCR (Disease Control Rate), which was 95.7% in the Apatinib group vs. an ORR of 2.2% and DCR of 58.7% in the placebo group. The median overall survival was not reached for Apatinib and was 29.9 months for placebo (hazard ratio, 0.42; 95% CI, 0.18–0.97; *p* = 0.04). The most common grade 3 or higher-level treatment-related adverse events in the Apatinib group were hypertension (34.8%], hand–foot syndrome (17.4%), proteinuria (15.2%), and diarrhea (15.2%)—none of which occurred in the placebo group. As yet, Apatinib has not obtained FDA and EMA approval. The main disadvantage of this trial was the low number of patients enrolled for a randomized trial; there were only 92 patients enrolled [52].

In DIRECTION, a Chinese multicentered double-blind randomized Phase III study, Donafenib, a novel multikinase inhibitor that targets Raf/MEK/ERK, VEGFR, and PDGFR, was evaluated for the treatment of RAIR DTC compared to placebo. The trial was positive for its primary end point, which was PFS. More precisely, Donafenib displayed a PFS of 12.9 months compared to 6.4 months for the placebo arm (HR0.39; *p* < 0.0001). Most common adverse effects were hypertension and palmoplantar erythrodysesthesia [53]. Again, Donafenib has not received FDA or EMA approval.

The efficacy and safety of Vandetanib was evaluated in VERIFY, a multicenter double-blind randomized Phase III trial. In VERIFY, Vandetanib, a TKI which inhibits VEGF2 and EGFR, was compared to placebo for the treatment of metastatic or locally advanced RAIR DTC. The trial showed a trend for a PFS survival benefit; however, this was not statistically significant (PFS 10 months vs. 5.7 months, HR 0.75, *p* = 0.08), which was the primary endpoint [54].

Beyond the Randomized Clinical Trials, there are many phase 2 studies evaluating different multikinase TKIs with encouraging results. There is evidence for the use of TKIs including Vandetanib, Sunitinib, Axitinib, and Pazopanib based on their respective phase II studies, but these drugs do not have FDA or EMA approval. The results can be found in Table 3. Hence, the only licensed multikinase TKIs for the treatment of RAIR remain Sorafenib and Lenvatinib for first-line treatment and Cabozatinib as subsequent-line treatment.

## 8. Other Targeted Therapies for DTC

### 8.1. Targeted Therapies for RET Fusions

RET fusions are present in about 10–20% of patients with papillary TCs [18]. Two different drugs have been approved for the treatment of RET, mutant progressive RAIR DTC, Selpercatinib (in Europe and in the United States), and Pralsetinib (only in the United States). In Europe, Selpercatinib is approved for patients who have already received sorafenib, Lenvatinib, or both, while in the United States, Selpercatinib is approved regardless of whether or not they have received previous treatment with sorafenib, Lenvatinib, or both.

Selpercatinib’s efficacy and safety was evaluated in the LIBRETTO-001 trial, which was an open-label, multicenter, phase 1/2 clinical trial. ORR among 19 patients with RET mutant RAIR DTC was 79% and 1-year PFS was 64%. The most common adverse events of grade 3 or higher were hypertension (21% of the patients), increased alanine aminotransferase levels (in 11%), increased aspartate aminotransferase levels (in 9%), hyponatremia (in 8%), and diarrhea (in 6%) [77].

In ARROW, a multi-center, open-label phase 1/2 trial, the efficacy and safety of Pralsetinib 400 mg daily was assessed in patients with RET-mutant medullary thyroid or RET fusion-positive DTC. Pralsetinib resulted in an ORR of eight out of nine patients (89%) with RET fusion-positive DTC. Common grades 3 and 4 treatment-related adverse events were hypertension (17%), neutropenia (13%), lymphopenia (12%), and anemia (10%). Serious treatment-related adverse events included pneumonitis (4%), while 4% of patients discontinued treatment due to toxicity [78].

### 8.2. Targeted Therapies for Patients with NTRK Fusions

Currently, two drugs have received approval for the treatment of solid tumors with NTRK fusions, Larotrectinib and Entrectinib. The efficacy of Larotrectinib, a highly selective TRK inhibitor targeting TRKA, TRKB, and TRKC, was examined in phase 1 and 2 clinical trials, in 17 different TRK fusion-positive cancer types, including TC. The ORR response reached 75% while adverse events were predominantly grade 1, with overall grade 3 or 4 adverse effects reaching 15% [79]. Based on these studies, in patients with TRK fusions, Larotrectinib received a tissue-agnostic drug approval.

In a pooled analysis from three phase I/II Larotrectinib clinical trials, there were 29 patients with TRK fusion-positive TC treated with Larotrectinib. Tumor histology was papillary (PTC) in 20 (69%) patients, follicular (FTC) in 2 (7%), and anaplastic (ATC) in 7 (24%) patients. Among 28 evaluable patients, ORR was 71%, CR in 7% of patients, partial response (PR) in 64%. ORR was 86% for PTC/FTC and 29% for ATC. The 24-month DoR, PFS, and OS rates were 81%, 69%, and 76%, respectively. Treatment-related adverse events were mainly grades 1–2 [80].

Entrectinib is another selective inhibitor targeting TRKA, TRKB, TRKC, ALK, and ROS1. Analysis of all phase 1/2 trials evaluating efficacy and safety of Entrectinib showed that among 54 adult patients, the ORR was 57%. The most common cancers treated with Entrectinib were sarcoma, NSCLC, mammary analogue secretory carcinoma, breast, thyroid, and colorectal. The most common grade 3 or 4 treatment-related adverse events were increased weight (10%) and anemia (12%). The most common serious treatment-related adverse events were nervous system disorders (4%). There were 13 patients with TC, 10 patients with PTC, and 3 patients non-PTC, with 1 patient having CR and 6 patients PR, with median duration of response being 13.2 months [81].

Both the FDA and EMA approvals for Larotrectinib and Entrectinib are for patients with metastatic, unresectable solid tumors harboring NTRK fusions in tumor agnostic indication that have no satisfactory treatment options or have progressed on standard-of-care treatment.

### 8.3. Targeted Therapies for BRAF Mutant DTC

V600E variant is the most common BRAF mutation found in DTC, and it is associated with a worse prognosis. In a phase 2 non-randomized, open label trial, the efficacy of Vemurafenib was evaluated for BRAF mutant RAIR DTC. In this study, ORR was 38.5%, with a median duration of response of 16.5 months, while SD was 35% for the cohort of patients that were treatment naive. In the cohort of patients who had received prior VEGFR inhibitors, ORR was 27.3% and 27.3% of patients had SD for at least 6 months. For this cohort, the median duration of response was 7.4 months, median PFS was 8.9 months and median OS was 14.4 months. Grade 3 or 4 adverse events were reported in 65% of the patients. Μost common grade 3 and 4 adverse events were squamous cell carcinoma of the skin (27%), lymphopenia (8%), and increased γ-glutamyl transferase (4%) [82].

In a randomized, open-label, multicenter Phase 2 clinical trial, a combined treatment of Dabrafenib (BRAF inhibitor) and Trametinib (MEK inhibitor) versus Dabrafenib alone was compared in patients with RAIR DTC. The primary endpoint was ORR by modified RECIST (minor response of −20% to −29%, partial and complete response as per standard RECIST criteria) within the first 24 weeks of therapy. A total of 53 patients were enrolled. The ORR (modified RECIST) was 42% with dabrafenib alone versus 48% with dabrafenib + trametinib, while ORR with RECIST 1.1 was 35% (9/26) with dabrafenib and 30% (8/27) with dabrafenib + trametinib. Hence the conclusion from this study was that the double blockade of dabrafenib and trametinib failed to improve outcomes compared to dabrafenib alone [83].

Selumetinib is a potent MEK1/2 inhibitor. Given that MEK is downstream from BRAF and given positive data for Selumetinib in BRAFV600E mutated thyroid cancer cell lines, a phase II clinical trial with RAIR patients was initiated. Of 32 evaluable patients, only one patient had a PR, while 21 patients had SD. It was noted that BRAF V600E-positive patients had a longer median PFS compared to wild type patients. The most common adverse events were fatigue, diarrhea, and rash. Out of 39 enrolled patients, 12 patients had dose reduction and 6 patients discontinued the treatment due to severe toxicity [73].

**Figure 1:**
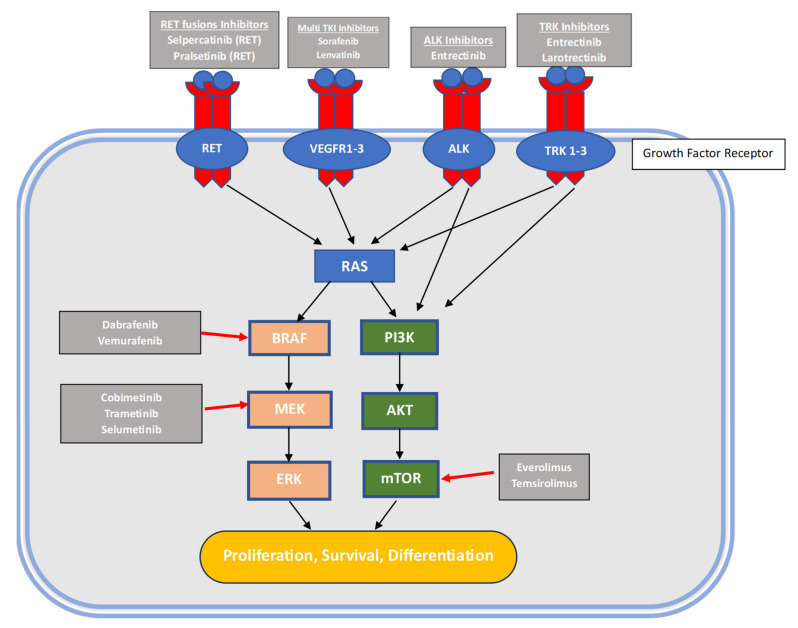
Molecular alterations and targeted agents.

In Figure 1, there is a graphic representation of molecular alterations found in patients with TC and the medications that target these mutations.

## 9. Management of Toxicity of Kinase Inhibitors

In Table 4, there is a description of TKIs currently used in clinical practice and the targeted protein that they inhibit. The most common adverse events (AEs) associated with TKI can be seen in Table 5. Multikinase TKIs (Sorafenib, Lenvatinib, and Cabozantinib) have a broader toxicity profile, as they inhibit multiple kinases. Serious adverse effects include gastro-intestinal symptoms with abdominal pain, diarrhea, nausea, vomiting, dehydration, and decreased appetite. They also include side effects relating to VEGFR inhibition with hypertension, bleeding, deep venous thrombosis, arterial thrombosis, pulmonary embolism and more rare side effects including reversible posterior leukoencephalopathy syndrome, proteinuria that can lead to nephrotic syndrome, renal failure, ejection fraction decrease, heart failure, QTc prolongation, perforation, impaired wound healing, and fistula formation.

Selpercatinib and Pralsetinib are more selective RET inhibitors with enhanced specificity for RET tyrosine kinase receptors (RTKs) over other RTK classes, however, they both inhibit to a lesser degree VEGFR, PDGFR, and FGFR, and therefore they can also give rise to a lesser degree hypertension, hemorrhagic events, impaired wound healing, hepatotoxicity, and interstitial lung disease/pneumonitis.

For NTRK inhibitors, serious adverse events grade 3–4 include liver test abnormalities, neutropenia, anemia, weight gain, myalgia, and neurological symptoms including gait disturbance, dizziness, and paresthesia.

Finally, Dabrafenib and Trametinib can give rise to pyrexia, anemia, leukopenia, infections, and renal impairment.

Serious adverse effects can affect quality of life, lead to hospitalization, and even death in a very small minority of patients. Hence, proactive management and patient education is very important, so that patients have clear instructions, for instance, on how to deal with diarrhea, hand–foot syndrome, and know how to monitor their blood pressure, aiming to prevent more serious toxicity. Prompt access to their physician and optimal treatment of side effects is essential [35].

Physicians can use available guidelines in conjunction with their own experience to manage TKI toxicity; the clinical experience of the treating physician in using multikinase TKIs is paramount. In our clinical practice, we commonly manage toxicity firstly by trying to improve symptom control using drugs (e.g., for hypertension, diarrhea, nausea, vomiting), practical advice for avoidance of hot spicy food (for stomatitis), use of emollients and exfoliation strategies for hand–foot syndrome. Secondly, if despite these measures symptoms persist or for toxicity that cannot be ameliorated by these methods (e.g., abnormal liver function tests, raised QTc, etc), dose interruption and then dose reduction is needed to control the toxicity and allow treatment continuation. Finally, the use of planned treatment breaks after dose reduction, e.g., using drug for 5 or 6 days per week can help to combat difficult to control toxicity.

## 10. Redifferentiation Therapy for Radioactive Iodine Refractory (RAIR) Differentiated Thyroid Cancer

As previously discussed in the molecular biology section, BRAF mutations dysregulate the capacity of follicular cells for iodine uptake and in fact BRAFV600E mutant thyroid cancers are often refractory to RAI. Thus, a hypothesis has emerged that targeting the BRAF V600E/MAPK pathway may reverse de-differentiation of TC cells and result in upregulation of the sodium iodide symporter (NIS) by inhibiting its negative regulators mediated by MEK and BRAF signaling. This strategy re-enables incorporation of iodine within the cancer cell and thus consists of the pretreatment with MEK and BRAF inhibitors, followed by RAI therapy [84]. This is currently studied in a number of different clinical trials, with promising initial results.

The first study by Ho et al. provided evidence for the hypothesis that inhibition of MEK can induce RAI uptake in RAIR DTC. In this study the MEK inhibitor Selumetinib was used in a cohort of 24 patients with RAIR DTC, treated at a dose of 75 mg twice a day. Selumetinib increased the iodine-124 uptake on PET-CT in all patients with N-RAS mutations (5) and in four out of the nine patients with BRAF mutation. Eight patients received RAI therapy, five patients had a partial response (one patient with BRAFV600E mutation), and three had stable disease. All patients showed a reduction in thyroglobulin levels. These results suggest that Selumetinib may be more effective in redifferentiation RAIR DTC in patients with NRAS compared to BRAFV600E mutations [85].

Another similar study aiming to investigate redifferentiation therapy was undertaken with vemurafenib, a BRAF inhibitor. In this pilot study of 10 patients who had BRAF V600E RAIR DTC, the main endpoint was the proportion of patients in whom vemurafenib would increase iodine uptake to warrant repeat RAI. Patients were evaluated with an iodine whole body scan after 4 weeks on vemurafenib 960 mg twice daily and then received RAI according to a pre-determined uptake threshold. Four out of ten patients showed increased iodine uptake after vemurafenib and received RAI, resulting in tumor regression 6 months after the treatment (40%). It was also noted that responders had higher baseline thyroglobulin values. Furthermore, tumor biopsies showed that vemurafenib inhibition of the MAPK pathway was associated with increased thyroid gene expression and RAI uptake. Hence, the conclusion from this study was that vemurafenib restores RAI uptake and efficacy in a subset of BRAF mutant RAIR patients, probably by upregulating thyroid-specific gene expression via MAPK pathway inhibition, and that higher baseline thyroglobulin values among responders suggest that tumor differentiation status may be a predictor of vemurafenib benefit [86].

A similar study was undertaken with another oral potent BRAF Inhibitor, Dabrafenib. In a small study of 10 patients with BRAF V600E DTC, iodine uptake was reassessed after treatment with Dabrafenib 150 mg twice daily for 25 days. A total of 60% of the patients had RAI uptake in prior non-radioactive iodine avid tissue. Six months after RAI and following dabrafenib, two patients showed PR and the remaining four patients had SD. The most common adverse effects were skin lesions, fatigue, electrolyte abnormalities, and palmar–plantar erythrodysesthesia [87].

In the MARAIODE, a multicenter phase 2 trial, the redifferentiation efficacy of dabrafenib in combination with trametinib for BRAF V600E-positive RAIR DTC was assessed. Following a baseline recombinant human (rh)TSH-stimulated diagnostic whole-body scan (dc1-WBS), dabrafenib and trametinib were given for 42 days. A second rhTSH-stimulated dc WBS (dc2-WBS) was done on day 28 and RAI was administered on day 35. The primary endpoint was the 6-month RECIST ORR. For patients with PR, a second RAI treatment was given. Abnormal iodine uptake was detected in 5%, 65%, and 95% of the dc1-WBS, dc2-WBS, and post-therapy scans, respectively. Hence, 20 out of 21 evaluable patients had increased iodine uptake after treatment with dabrafenib plus trametinib. The primary endpoint ORR at 6 months was achieved in 38% of patients, SD in 52%, and PD in 10%. Ten patients received a second treatment course: one complete response and sux PRs were observed at 6 months. The median PFS was not reached, with 12- and 24-month PFSs of 82% and 68%. Adverse events occurred in 96% of the patients, with grade 3–4 toxicity in 33% of patients [88].

More recent clinical trials have adopted more personalized approaches to patients with redifferentiation therapy according to BRAF status, mutated, or wild type and RAS-mutated tumors. With the rationale that results with differentiation therapy were unsatisfactory in BRAFV600E-mutant RAIR, Weber and colleagues tested redifferentiation therapy through genotype-guided MAPK modulation, offering patients with BRAF-Mutations trametinib + dabrafenib, while patients with wildtype BRAF-WT received trametinib for 21 ± 3 days. Redifferentiation was assessed by 123I-scintigraphy. In case of restored radioiodine uptake, 124I-guided 131I therapy was performed. Redifferentiation was achieved in 7 out of 20 (35%) patients in total, with 2 out of 6 (33%) in the BRAF positive cohort, and 5 out of 14 (36%) in the BRAF-WT arm. Thyroglobulin decline was seen in 57% (4/7) of the patients, while PR by RECIST was documented in 14% (1/7) and SD in 71% (5/7). A peak standardized uptake value (SUV peak) of <10 on FDG-PET was associated with successful redifferentiation (*p* = 0.01). Hence, in conclusion, successful redifferentiation was achieved in about one third of patients in each arm, with a suggestion that low tumor glycolytic rate assessed by FDG-PET is predictive of redifferentiation success [89].

A similar principle applied to the study by Iravani et al., where patients with NRAS mutations received the MEK inhibitor trametinib, while patients with BRAFV600E mutations were treated with combination BRAF and MEK inhibitors, with four out of six patients achieving restoration of RAI uptake and partial response to RAI treatment [90].

In Table 6, all the clinical trials examining redifferentiation of RAIR TC according to clinicaltrials.gov are included.

## 11. Targeted Therapies of the PI3K/Akt Pathway

In addition to the RET-RAS-RAF-MAPK signaling pathway, the PI3K (phosphatidylinositol 3-kinase)/Akt pathway is involved in thyroid carcinogenesis, DTC dedifferentiation, angiogenesis, cell proliferation, progression, and metastasis reported in aggressive PTC [69]. In view of the above observations, a number of studies have been undertaken with Everolimus, which is an mTOR inhibitor.

In the first study by Lim et al., 38 patients with advanced thyroid cancer of all histologic subtypes were treated with Everolimus 10 mg od. There was a 5% PR and 45% durable SD. The median PFS was 47 weeks [91].

In a second study, Hanna et al. enrolled 40 patients with follicular cell-origin thyroid cancer, 33 patients with DTC, and 7 patients with ATC to receive 10 mg of Everolimus. A total of 72.5% of patients with follicular cell-origin cancer had SD and only 5% had a PR. For the DTC cohort, median PFS was 12.9 months with a 2-year PFS of 23.6% and 2-year OS was 73.5% [92].

Finally, in another phase II study by Schneider et al., 28 patients with progressive metastatic or locally advanced RAIR DTC received Everolimus 10 mg orally once daily. Seventeen patients (65%) showed SD, of which fifteen (58%) showed SD > 24 weeks. No CR or PR was observed. Median PFS and OS were 9 and 18 months, respectively. Toxicity was predominantly grade 1/2 consistent with the known toxicity profile of Everolimus and included anemia (64%), cough (64%), stomatitis (61%), and hyperglycemia (61%), with 46% of the patients requiring dose reduction. The presence of somatic gene variants related to mTOR signaling did not clearly correlate with outcomes [93].

## 12. Optimal First-Line Therapy and Sequencing of Therapies in Patients with Targetable Genetic Alterations

The optimal choice and sequence of multikinase and selective tyrosine kinase inhibitors in RAIR DTC is not clear. The ESMO guidelines suggest that treatment should be continued until disease progression, unacceptable toxicity, or the patient’s request to stop, and that previous TKI therapy is not a contraindication to subsequent use of a different TKI. The ESMO guidelines also suggest that in the presence of single site progression, locoregional treatment can be used for local control without discontinuing the TKI [43].

Regarding the optimal first-line treatment choice, between the multikinase inhibitors Lenvatinib and Sorafenib, the authors’ practice is governed by the need for cytoreduction in conjunction with the performance status and comorbidities of the patient. In patients with heavy disease burden and who are symptomatic, in which case urgent cytoreduction is needed, Lenvatinib is the preferred treatment. For patients who are not symptomatic, with lower disease burden, who are frailer, especially with cardiac co-morbidity, and where tolerance of the treatment is more important as opposed to obtaining a radiological response, in those patients Sorafenib may be the preferred choice.

For patients with BRAF V600E RAIR DTC, treatment options for first-line treatment would lie between the multikinase TKIs Lenvatinib or Sorafenib, versus targeted therapy with Dabrafenib and Trametinib based on the FDA tumor agnostic approval. When considering the rationale of providing the most effective treatment upfront and extrapolating from data from the registration trials, based on the higher Overall Response Rate (ORR) of Lenvatinib in the SELECT trial compared to the ORR for combined BRAF and MEK inhibitors in a number of different phase II studies, it can be argued that Lenvatinib should be offered as first-line treatment, reserving the use of Dabrafenib and Trametinib further down the line. This in fact reflects the FDA approval wording, which is for metastatic solid tumors with BRAF V600E mutation that have progressed following prior treatment and have no satisfactory alternative treatment options. However, the emerging evidence of use of these agents in causing redifferentiation of RAIR, and as a result facilitating repeat RAI treatment, needs to be acknowledged. Results of ongoing redifferentiation trials are awaited with the potential to change the current treatment approach in the near future.

Following the same rationale to offer the more effective treatment upfront, it could be argued that RET and NTRK inhibitors should be offered upfront as their registration trials show superior ORR and PFS compared to the outcomes seen with the SELECT and DECISION registration clinical trials for Lenvatinib and Sorafenib. There are, however, differences in the approvals for these drugs; in Europe, Selpercatinib is approved as second-line treatment after Sorafenib, Lenvatinib, or both, while in the United States, Selpercatinib or Pralsetanib can be used either as first or second-line therapy. Equally, for the NTRK inhibitors Larotrectinib and Entrectinib, the current FDA and EMA approvals are for patients with RAIR, with no satisfactory alternative treatments for those whose cancer has progressed following treatment, hence, the use of NTRK inhibitors should be reserved for post-multikinase TKI use. Finally, Cabozatinib can be offered after the first-line Sorafenib or Lenvatinib, as per licensed indication.

## 13. Other Therapeutic Options

### 13.1. Peptide Receptor Radionuclide Therapy (PRRT)

Another therapeutic option for RAIR patients is the use of radiolabeled somatostatin receptor (SSTR) analogs, i.e., Peptide Receptor Radionuclide Therapy (PRRT) for the subset of patients that express somatostatin receptors. To do this, firstly, PET/CT imaging is undertaken with radiolabeled somatostatin receptor (SSTR) analogs, such as 68Ga-DOTATATE, and then patients with RAIR DTC and positive 68Ga-DOTATATE uptake can be considered for Peptide Receptor Radionuclide Therapy (PRRT) with either Lutetium or Yttrium.

A number of small pilot studies of patients with progressive RAIR DTC have provided the proof of concept [94,95]. In the Versari study, from 41 patients with progressive RAIR and following (68)Ga-DOTATOC PET/CT to identify SSTR expression, with positive results in more than half of the cases, about one third of patients were eligible for PPRT. In this study, 11 out of 41 patients were treated with PRRT receiving a fractionated injection (90)Y-DOTATOC. PRRT-induced disease control in 7/11 patients (two PR and five SD) with a duration of response of 3.5–11.5 months. Main PRRT adverse events were nausea, asthenia, and transient hematologic toxicity, while one patient experienced permanent renal toxicity [95].

Overall, the outcomes of PPRT from these small pilot studies in somatostatin receptor-positive patients, relating to ORR and duration of response, is similar to treatment with TKIs, however, with a better safety and toxicity profile. Hence, PRRT is potentially an effective and safe treatment option for somatostatin receptor-positive RAIR DTC [95].

### 13.2. Immunotherapy for RAIR DTC

DTC has been considered to be poorly immunogenic due to a low Tumor Mutation Burden (TMB), with data from the TCGA from PTC samples showing an average of 0.41 nonsynonymous mutations (mut)/Mb, which is a low TMB compared to other histologies [10]. However, DTC is infiltrated by immune cells, including NK, tumor-associated macrophages, mast cells, dendritic cells, B, and T lymphocytes, often with expression of CTLA-4 and PD-L1 [96]. The intra-tumoral immune cell density correlates with BRAF V600E mutation and low thyroid differentiation scores, while PD-L1 positivity has been shown to correlate with lymph node metastasis, extra-nodal invasion, tumor recurrence, and poor survival in thyroid cancer patients [97]. The latter observation serves as the basis for the hypothesis to target the PD-1/PD-L1 pathway in DTC.

Clinical trials with single agent checkpoint inhibitors, however, have shown modest results so far. In the phase Ib KEYNOTE-028 trial, from 51 patients with DTC screened, 71% (*n* = 36) were PD-L1 positive, and finally 22 patients were treated with Pembrolizumab, with an ORR of 9% (two patients with PTC with response duration of 20 and 8 months). Fifty-seven percent of the patients with FTC and sixty percent of patients with PTC had a SD with a median PFS of 7 months. PFS rates at 6 and 12 months were 59 and 36% [98].

Similar results were seen with combination immunotherapy with nivolumab and ipilimumab. This combination was tested in a phase II study in three different cohorts: RAIR DTC (*n* = 32, including 4 patients with poorly differentiated TC), ATC (10 patients) and MTC (7 patients). ORR for each of the three cohorts was 9.4% [99].

Of great interest are the results of the KEYNOTE-158 trial, which suggest that TMB could identify patients more likely to respond to immunotherapy. KN -158 was a phase II multicohort trial that evaluated pembrolizumab as monotherapy in different cohorts of rare cancers, with cohort I enrolling patients with PTC and FTC. Median TMB of the cohort was 1.7 mut/Mb with only six patients (2.7%) qualifying as TMB-high (>12 mut/Mb). In 80 patients with evaluable disease, ORR was 100% (2/2) for patients with high TMB and only 3.8% (3/78) for patients with low TMB [100].

More promising results have been seen with the combination of TKIs and checkpoint inhibitors, and this strategy is being actively tested in prospective clinical trials [75]. In a single-arm phase II trial with 29 evaluable patients with RAIR DTC, the combination of Lenvatinib with pembrolizumab resulted in an ORR of 62% (with no CR) and 35% SD. Median PFS was not reached, while 12 months PFS was 74% [101].

A different cohort of the same study evaluated the addition of Pembrolizumab to Lenvatinib in patients who had already progressed on Lenvatinib with initial promising results. Of 20 evaluable patients, 3 patients (15%) had a PR and 17 (85%) had SD. Median PFS was 12.6 months, while PFS at 12 months was 56%. The treatment was tolerated well, with 36% of patients developing grade 3 AEs and no grade 4/5 AEs [102].

Finally in Table 7 there is a list of ongoing clinical Trials for patients with RAIR DTC.

## 14. Conclusions

Care should be exercised regarding the timing of the initiation of systemic therapy for patients with RAIR, as current treatments are non-curative, and they are aimed at palliation of symptoms and prolongation of survival. These treatments are essentially life-long, and are associated with significant toxicity; at the same time, many patients with RAIR may be asymptomatic with a slow disease tempo. Hence, many patients may not need to start systemic therapy immediately and instead a period of observation would be indicated prior to initiation of therapy.

Multikinase tyrosine kinase inhibitors provide an effective and promising treatment option for patients with RAIR thyroid cancer, with Lenvatinib and Sorafenib having become the standard first-line systemic therapies, and should be used by oncologists with experience in their use and for management of toxicity. Cabozantinib is an option to treat patients with RAIR following progression on Lenvatinib or Sorafenib.

All patients with RAIR DTC should undergo molecular testing to look at potentially actionable genetic alterations with next-generation sequencing (NGS) to facilitate targeted treatment and entry into clinical trials. For patients with RET fusions, Selpercatinib and Pralsetinib are especially effective, and can be used either in the first- or second-line settings.Equally, for patients harboring NTRK fusions, Larotrectinib and Entrectinib can be used. Targeted therapies can also be considered for patients with BRAF V600E mutations after prior TKI use.

In the near future the prospect of redifferentiation therapy, aiming to restore the ability of thyroid cells to concentrate iodine and receive again RAI is a very exciting and promising strategy, and further clinical studies are currently underway with the potential to change the treatment paradigm for patients with RAIR. Potentially, redifferentiation therapy can come in the near future prior to the use of TKIs after a short period of targeted therapy.

Also in the near future, there is promise in the use of Peptide Receptor Radionuclide Therapy (PPRT) for somatostatin receptor-positive RAIR DTC, again with the potential of this treatment coming ahead from the use of TKIs, as there may be less toxicity than with TKIs.

Finally, there are many ongoing clinical trials with novel therapies, with trials of immune checkpoint inhibitors in combination with TKIs in progress, aiming to produce both high response rates with long duration of responses.

## Figures and Tables

**Table 1:** Genetic alterations and different types of TCs [19,29,35].

	BRAF	RET	NTRK	PIK3CA	RAS	PAX8/PPARγ	PTEN	ALK
PTC	50–60%	10%	2%	2%	10–20%		2%	1%
FTC				1–10%	40–50%	30–35%	<10%	
PDTC	5–35%			2–10%	20–40%			4–9%
MTC		100% (familial) 50% (sporadic)			40% (sporadic)			
ATC	10–50%	4%		10–20%	20–40%		5–15%	

**Table 2:** Phase 3 trials addressing the utilization of TKIs for the treatment of RAIR DTC.

NCT	Title	Description	Intervention	Outcomes	Results	Reference
NCT00984282	Sorafenib in radioactive iodine-refractory, locally advanced or metastatic diff erentiated thyroid cancer: a randomised, double-blind, phase 3 trial	Phase 3, Randomized, Double-Blind	Sorafenib vs. Placebo	Primary: PFS Secondary: Safety	PFS: 10.8 months (Sorafenib) vs. 5.8 (Placebo) HR 0.59 *p* < 0.0001	[48]
NCT01321554	Lenvatinib versus Placebo in Radioiodine- Refractory Thyroid Cancer	Phase 3, Randomized, Double-Blind	Lenvatinib vs. Placebo	Primary: PFS Secondary: ORR, Safety	PFS: 18.3 (Lenvatinib) vs. 3.6 (Placebo)HR 0.21 *p* < 0.001, ORR: 64.8% vs. 1.5%	[49]
NCT02966093	A Multicenter, Randomized, Double-Blind, Placebo-Controlled, Phase 3 Trial of Lenvatinib (E7080) in 131 I-Refractory Differentiated Thyroid Cancer in China	Phase 3, Randomized, Double blind	Lenvatinib vs. placebo	Primary: PFS Secondary: ORR, OS, Safety	PFS: 23.9 months (Lenvatinib) vs. 3.7 (placebo) *p* < 0.0001 ORR: 69.9 (Lenvatinib) vs. 0 (placebo)	[50]
NCT03690388	A Phase 3, Randomized, Double-Blind, Placebo-Controlled Study of Cabozantinib (XL184) in Subjects with Radioiodine-Refractory Differentiated Thyroid Cancer Who Have Progressed After Prior Vascular Endothelial Growth Factor Receptor (VEGFR) -Targeted Therapy	Phase 3, Multicenter, Randomized, Double-Blind, Placebo controlled	Cabozantinib vs. placebo	Primary: PFS Secondary: ORR	PFS: mPFS not Reached in experimental group vs. 1.9 months in plaebo. ORR: 15% vs. 0%	[51]
NCT03048877	Efficacy of Apatinib in Radioactive Iodine-refractory Differentiated Thyroid Cancer	Phase 3, Randomized, Double blind	Apatinib vs. Placebo	Primary: PFS	PFS: 22.4 months (Apatinib) vs. 4.5 months (Placebo) HR 0.26, *p* < 0.001	[52]
NCT03602495	A Multicenter, Randomized, Double-blind, Placebo-controlled, Phase 3 Study of Donafenib in Patients with Radioiodine-Refractory Differentiated Thyroid Cancer	Phase 3, Randomized, Double-Blind	Donafenib vs. Placebo	Primary: PFS Secondary: OS, ORR, DCR, TTP	Interim Analysis PFS: 12.9 months (Donafenib) vs. 6.4 (Placebo) *p* < 0.0001, ORR 23.3 (Donafenib) vs. 1.7 (Placebo)	[53]
NCT01876784	Evaluation of Efficacy, Safety of Vandetanib in Patients with Differentiated Thyroid Cancer (VERIFY)	Phase 3, Randomized, Double Blind	Vandetanib vs. Placebo	Primary: PFS	PFS: 10 months (Vandetanib) vs. 5.7 (Placebo) HR 0.75 (*p* = 0.08)	[54]

RAI: Radioactive Iodine, CRRR: Complete Response Rate, CoRR: Confirmed Response Rate, AE: Adverse Effects, PFS: Progression Free Survival, OS: Overall Survival, ORR: Objective Response Rate, TTP: Time to Progression, TG: Thyroglobulin, DOR: Duration of Response, BRR: Biochemical Response Rate, DCR: Disease Control Rate, BOR: Best Overall Response, CBR: Control Benefit Rate.

**Table 3:** Phase 2 trials of TKIs for the treatment of RAIR DTC.

NCT	Title	Description	Intervention	Outcomes	Results	Reference
NCT02702388	A Trial of Lenvatinib (E7080) in Subjects with Iodine-131 Refractory Differentiated Thyroid Cancer to Evaluate Whether an Oral Starting Dose of 18 Milligram (mg) Daily Will Provide Comparable Efficacy to a 24 mg Starting Dose, But Have a Better Safety Profile	Phase 2, Randomized, Double Blind	Lenvatinib 24 mg vs. Lenvatinib 18 mg	Primary: ORR, Rate of Grade > 3 AE Secondary: PFS, PFS2, Safety, Time of First Dose reduction, Number of Dose Reductions	ORR: 57.3 (24 mg) vs. 40.3 (18 mg) Rate of Grade > 3 AE: 61.3 (24 mg) vs. 57.1 (18 mg) PFS: Not reached (24 mg) vs. 24.4 months (18 mg)	[58]
NCT00519896	Phase II study of daily sunitinib in FDG-PET-positive, iodine-refractory differentiated thyroid cancer and metastatic medullary carcinoma of the thyroid with functional imaging correlation	Phase 2, Single Group, Open Label	Sunitinib	Primary: ORR Secondary: Safety and Toxicity, TTP	ORR: 31% TTP: 12.8 (8.9–N/A)	[60]
NCT00654238	Phase II Trial of Sorafenib (Nexavar) in Patients with Advanced Thyroid Cancer	Phase 2, Single Group, Open Label	Sorafenib	Primary: ORR Secondary: PFS	ORR: 36.4 (DTC) PFS: 77 weeks (60–96)	[61]
NCT01263951	Study of Everolimus and Sorafenib in Patients with Advanced Thyroid Cancer Who Progressed on Sorafenib Alone	Phase 2, Single Group, Open Label	Everolimus plus Sorafenib	Primary: PFS Secondary: CBR	PFS: 13.7 months (7.15–24.75)	[62]
NCT02870569	A Multicenter, Randomized, Open-Label, Phase 2 Trial of Donafenib in 131I-Refractory Differentiated Thyroid Cancer	Phase 2, Parallel Study of two different doses of Donafenib, Open Label	Donafenib 200 mg or Donafenib 300 m	Primary: ORR Secondary: OS, PFS, Safety, DCR	ORR: 12.5% (200 mg), 13.3% (300 mg) PFS: 9.44 (200 mg) months vs. 14.98 months (300 mg) *p* = 0.351)	[63]
NCT00537095	Vandetanib in locally advanced or metastatic differentiated thyroid cancer: a randomised, double-blind, phase 2 trial	Phase 2, Randomized, Double-Blind	Vandetanib vs. Placebo	Primary: PFS	PFS: 11.1 months (Vandetanib) vs. 5.9 (Placebo) HR 0.62, *p* = 0.008	[64]
NCT00510640	Thyroid Cancer and Sunitinib (THYSU)	Phase 2, Open Label	Sunitinib	Primary: ORR Secondary: Safety	ORR:22% DTC	[65]
NCT02614495	Study of Sulfatinib in Treating Advanced Medullary Thyroid Carcinoma and Iodine-refractory Differentiated Thyroid Carcinoma	Phase 2, Open Label	Surufatinib	Primary: ORR Secondary: Safety, DCR, PFS	ORR: 21.7–33.3% DTC PFS: 11.1 months DTC	[66]
NCT00625846	Pazopanib Hydrochloride in Treating Patients with Advanced Thyroid Cancer	Phase 2, Open Label	Pazopanib	Primary: ORR Secondary: Safety, PFS, DoR	ORR: 49% DTC	[67]
NCT00094055	Study of the Anti-angiogenesis Agent AG-013736 in Patients with Metastatic Thyroid Cancer	Phase 2, Open Label	Axitinib	Primary: ORR Secondary: PFS, DoR, OS	ORR: 38.3% PFS: 459 days DoR: 625 days OS: 1068 days	[68]
NCT01270321	Pasireotide and Everolimus in Adult Patients with Radioiodine-Refractory Differentiated and Medullary Thyroid Cancer	Phase 2, 3 Arm, Open Label	Everolimus, Pasireotide	Primary: ORR	No PR according to RECIST 1.1	[69]
NCT02084732	Safety and Efficacy of Sorafenib in Patients with Advanced Thyroid Cancer: a Phase II Clinical Study	Phase 2, Open Label	Sorafenib	Primary: ORR Secondary: Safety	ORR: 35.7%	[70]
NCT01025453	Phase II Study Evaluating the Combination of Temsirolimus and Sorafenib in the Treatment of Radioactive Iodine Refractory Thyroid Cancer	Phase 2, Open Label	Temsirolimus and Sorafenib	Primary: ORR Secondary: Safety	ORR: 23.7%	[71]
NCT01964144	An Open-label, Multicenter, Phase II Study of Dovitinib in Advanced Thyroid Cancer	Phase 2, Open Label	Dovitinib	Primary: ORR	ORR: 20.5%	[72]
NCT00559949	Phase 2 Study of Selumetinib Hydrogen Sulfate in Iodine-131 Refractory Papillary Thyroid Carcinoma and Papillary Thyroid Carcinoma with Follicular Elements	Phase 2, Open Label	Salumetinib	Primary: ORR Secondary: PFS, Safety, OS	ORR: 3.1% PFS: 32 weeks	[73]
NCT01811212	Phase II Study of Cabozantinib in Patients with Radioiodine-Refractory Differentiated Thyroid Cancer Who Progressed on Prior VEGFR-Targeted Therapy	Phase 2, Open Label	Cabozatinib	Primary: ORR Secondary: Bone Turnover, DoR, Safety	ORR: 40% DoR: 11.3 months	[74]
NCT02586337	A Randomized, Double-blind, Placebo-controlled, Multicenter Clinical Trial to Compare the Efficacy and Safety of Anlotinib Versus Placebo in Patients with 131I-Refractory Differentiated Thyroid Cancer (ALTER01032)	Phase 2 Randomized, Double blind	Anlotinib vs. Placebo	Primary: PFS	Median PFS 40.5 months vs. placebo 8.4 months, HR = 0.21, *p* < 0.001],	[75]
NCT01208051	A multicenter, open label, randomized, phase II study of cediranib with or without lenalidomide in iodine 131-refractory differentiated thyroid cancer	Phase 2, Randomized, Open Label,	Cediranib vs. Cediranib with Lenalidomide	Primary: PFS Secondary: ORR, DoR, Safety	Median PFS 14.8 months (Cediranib) vs. 11.3 months (Cediranib plus Lenalidomide)	[76]

RAI: Radioactive Iodine, Complete Response Rate, CoRR: Confirmed Response Rate, AE: Adverse Effects, PFS: Progression Free Survival, OS: Overall Survival, ORR: Objective Response Rate, TTP: Time to Progression, TG: Thyroglobulin, DOR: Duration of Response, BRR: Biochemical Response Rate, DCR: Disease Control Rate, BOR: Best Overall Response, CBR: Control Benefit Rate.

**Table 4:** Tyrosine kinase inhibitors and targets they inhibit.

Drug	VEGFR-1	VEGFR-2	VEGFR-3	c-KIT	RET	PDGFR	FGFR	TRK	OTHER
Lenvatinib	+	+	+	+	+	+	+	−	RET-KIF5B
Sorafenib	−	+	+	+	+	+	−	−	RAF, FLT3
Cabozantinib	−	+	−	+	+	−	−	−	FLT3, MET, AXL, TIEZ, RET-KIF5B
Larotrectinib	−	−	−	−	−	−	−	+	-
Entrectinib	−	−	−	−	−	−	−	+	ALK, ROS1
Selpercatinib	−	−	−	−	+	−	−	−	-
Pralsetinib	−	−	−	−	+	−	−	−	-
Vemurafenib	−	−	−	−	−	−	−	−	BRAFV600E
Dabrafenib	−	−	−	−	−	−	−	−	BRAFV600E

**Table 5:** Most common adverse events of TKIs used in common clinical practice for the treatment of RAIR DTC.

**Sorafenib**	Hand–foot syndrome, diarrhea, nausea, vomiting, hypertension, bleeding, arthralgia, increased amylase/lipase, rash and dry skin.
**Lenvatinib**	Hypertension, diarrhea, fatigue, proteinuria, hand–foot syndrome decreased weight, nausea, vomiting, stomatitis, dysphonia.
**Cabozantinib**	Diarrhea, hand–foot syndrome, hypertension, fatigue, decreased appetite, nausea, rise in transaminases.
**Pralsetinib**	Constipation, diarrhea, fatigue, neutropenia, anemia, hypertension transaminases increase, musculoskeletal pain, pneumonia, pneumonitis.
**Selpercatinib**	Increased transaminases, vomiting, constipation, diarrhea, nausea, rise QTC, hypertension, bleeding, fatigue, oedema.
**Entrectinib**	Fatigue, constipation, oedema, dizziness, diarrhea, nausea, oedema, dysesthesia, dyspnea, anemia, increased weight, pain, cognitive disorders, cough, and pyrexia.
**Larotrectinib**	Increased transaminases, vomiting, constipation, diarrhea, myalgia, fatigue, anemia, decreased neutrophil count, dizziness, paresthesia.
**Dabrafenib and Trametinib**	Pyrexia, anemia, decreased appetite, fatigue, nausea, infections, pneumonia, pleural effusion, renal impairment, leukopenia.

**Table 6:** Clinical trials of redifferentiation therapy (to increase RAI uptake in DTC).

NCT	Title	Description	Intervention	Outcomes	Results	Reference
NCT00970359	Reacquisition of Radioactive Iodine (RAI) Uptake of RAI-Refractory Metastatic Thyroid Cancers by Pretreatment with the Selective MEK Inhibitor AZD6244	Single Group, Open Label	Selumetinib	Primary: Number of patients who have increased radioiodine uptake, ORR Secondary: TG level change	Primary: 12/20 increased uptake. 8 patients had RAI with 5/8 PR and 3/8 SD. Secondary: All patients had decrease in TG (mean reduction 89%)	[84]
NCT02145143	Enhancing Radioiodine (RAI) Incorporation Into BRAF Mutant, RAI-Refractory Thyroid Cancers with the BRAF Inhibitor Vemurafenib: A Pilot Study	Pilot study	Vemurafenib	Primary: Increased uptake and Response to RAI.	Primary: 4/10 patients had increased uptake and received RAI, resulting in 6 month regression	[85]
NCT01534897	Re-differentiation of Radioiodine-Refractory BRAF V600E-mutant Papillary Thyroid Carcinoma with GSK2118436 (Dabrafenib)	Single Group, Open Label	Dabrafenib	Primary: Number of patients who have increased radioiodine uptake Secondary: Safety, ORR, TG level	Primary: 6/10 patients increased uptake. Secondary: 2/6 patients treated with RAI PR, and 4/6 SD.	[86]
NCT03244956	Efficacy of MEK (Trametinib) and BRAFV600E (Dabrafenib) Inhibitors with Radioactive Iodine (RAI) for the Treatment of Refractory Metastatic Differentiated Thyroid Cancer (MERAIODE)	Phase 2, Non-Randomized, Open Label	Dabrafenib plus Trametinib or Trametinib (Depends on BRAF mutation status)	Primary: ORR at 6 months	ORR: 38.8%, SD in 52% and PD in10%.	[87]
NCT04619316	Enhancing Radioiodine Incorporation Into Radio Iodine Refractory Thyroid Cancers with MAPK Inhibition (ERRITI)	Phase 2, Open-Label	Trametinib (BRAF WT), Trametinib plus Dabrafenib (BRAF MT)	Primary: Redifferentiation rate	7/20 patients, 2/6 BRAF MT, 5/14 BRAF WT	[88]

RAI: Radioactive Iodine, ORR: Objective Response Rate, TG: Thyroglobulin, DOR: Duration of Response.

**Table 7:** Selected ongoing clinical trials addressing the use of TKIS for the treatment of RAIR DTC.

NCT	Title	Description	Intervention	Outcomes	Results	Reference
NCT02973997	Lenvatinib and Pembrolizumab in Differentiated Thyroid Cancers (DTC)	Phase 2, Single Group, Open Label	Lenvatinib and Pembrolizumab	Primary: CRRR, Confirmed CoRR Secondary: Incidence AE, PFS, OS	Pending	Clinicaltrials.gov (accessed 10 March 2023) [54]
NCT04554680	Clinical Trial in RAI-Refractory Thyroid Carcinoma Evaluating BRAF and MEK Blockade for Re-differentiation Therapy	Phase 2, Single Group, Open Label	Dabrafenib plus Trametinib	Primary: Proportion of patients with at least one iodine avid lesion Secondary: PFS, Best Tumor Response, Change in TG levels, AE	Pending	Clinicaltrials.gov (accessed 10 March 2023) [54]
NCT04061980	Encorafenib and Binimetinib with or Without Nivolumab in Treating Patients with Metastatic Radioiodine Refractory BRAF V600 Mutant Thyroid Cancer	Phase 2,Randomized, Open Label	Encorafenib and Binimetinib vs. Encorafenib, Binimetinib and Nivolumab	Primary: ORR Secondary: PFS, OS, DOR	Pending	Clinicaltrials.gov (accessed 10 March 2023) [54]
NCT04952493	Anlotinib or Penpulimab in Combination with RAI for DTC	Phase 2, Randomized, Open Label	Anlotinib plus RAI vs. Penpulimab plus RAI	Primary: ORR Secondary: BRR, DCR, PFS	Pending	Clinicaltrials.gov (accessed 10 March 2023) [54]
NCT03914300	Testing the Combination of Cabozantinib, Nivolumab, and Ipilimumab (CaboNivoIpi) for Advanced Differentiated Thyroid Cancer	Phase 2, Single Group, Open Label	Cabozatinib plus Ipilimumab plus Nivolumab	Primary: ORR Secondary: DOR, Safety	Pending	Clinicaltrials.gov (accessed 10 March 2023) [54]
NCT03573960	A Study to Evaluate the Safety and Efficacy of Lenvatinib in Participants with Refractory Differentiated Thyroid Cancer	Phase 4, Single Group, Open Label	Lenvatinib	Primary: Percentage of >G2 AE, Number of Dose Reduction, Median to Dose Reduction Secondary: ORR, PFS, Percentage of G1 AE	Pending	Clinicaltrials.gov (accessed 10 March 2023) [54]
NCT04560127	A Single-arm, Non-randomized, Single-center Study to Evaluate Camrelizumab in Combination with Apatinib in Patients with Radioactive Iodine-refractory Differentiated Thyroid Cancer	Phase 2, Single Group, Open Label	Camrelizumab plus Apatinib	Primary: PFS Secondary: ORR, OS, DCR, DoR, Safety	Pending	Clinicaltrials.gov (accessed 10 March 2023) [54]
NCT02041260	A Phase II Trial of Cabozantinib for the Treatment of Radioiodine (RAI)-Refractory Differentiated Thyroid Carcinoma (DTC) in the First-line Setting	Phase 2, Single Group, Open Label	Cabozantinib	Primary: number of AE	Pending	Clinicaltrials.gov (accessed 10 March 2023) [54]
NCT05745363	Phase Ib Clinical Trial to Evaluate the Efficacy and Safety of AL2846 Capsule in Iodine-resistant Differentiated Thyroid Cancer with Previous TKI Treatment Failure	Phase 1 and 2, Single Group, Open Label	AL2846 capsule	Primary: ORR Secondary: PFS, DCR, DoR, OS, Safety	Pending	Clinicaltrials.gov (accessed 10 March 2023) [54]
NCT03469011	A Study to Try to Bring Back Radioiodine Sensitivity in Patients with Advanced Thyroid Cancer.	Phase 1, Single Group, Open Label	Imatinib	Primary: Increment of RAI uptake	Pending	Clinicaltrials.gov (accessed 9 July 23) [54]
NCT01396733	Redifferentiation Therapy Using Alpha Lipoic Acid in Thyroid Cancer (RALT)	Phase 2, Randomized, Open Label	Alpha-lipoic acid (RALT)	Primary: Increment of Iodine uptake	Pending	Clinicaltrials.gov (accessed 9 July 23) [54]
NCT04554680	Clinical Trial in RAI-Refractory Thyroid Carcinoma Evaluating BRAF and MEK Blockade for Re-differentiation Therapy	Phase 2, Single Group, Open Label	Dabrafenib and Trametinib	Primary: Rate of patient with RAI uptake Secondary: PFS, ORR, TG level	Pending	Clinicaltrials.gov (accessed 9 July 23) [54]
NCT05507775	Digoxin for the Reinduction of Radioiodine Uptake in Metastatic or Locally Advanced Non-medullary Thyroid Carcinoma (DIGUP-TC)	Single Group, Open Label	Digoxin	Primary: Number of patients who have increased radioiodine uptake, ORR Secondary: safety	Pending	Clinicaltrials.gov (accessed 9 July 23) [54]
NCT02145143	Enhancing Radioiodine (RAI) Incorporation Into BRAF Mutant, RAI-Refractory Thyroid Cancers with the BRAF Inhibitor Vemurafenib: A Pilot Study	Single Group, Open Label	Vemurafenib	Primary: DoR Secondary: ORR, safety	Pending	Clinicaltrials.gov (accessed 9 July 23) [54]
NCT05733013	A Study to Collect Information About the Use of Redifferentiating Medications as a Standard Treatment for Thyroid Cancer	Prospective, Observational Study	Observational: Known re-differentiation	Primary: Safety	Pending	Clinicaltrials.gov (accessed 9 July 23) [54]
NCT05783323	Larotrectinib to Enhance RAI Avidity in Differentiated Thyroid Cancer	Single Group, Open Label	Larotrectinib	Primary: Number of patients with complete pulmonary structural response	Pending	Clinicaltrials.gov (accessed 9 July 23) [54]

RAI: Radioactive Iodine, CRRR: Complete Response Rate, CoRR: Confirmed Response Rate, AE: Adverse Effects, PFS: Progression Free Survival, OS: Overall Survival, ORR: Objective Response Rate, TTP: Time to Progression, TG: Thyroglobulin, DOR: Duration of Response, BRR: Biochemical Response Rate, DCR: Disease Control Rate, BOR: Best Overall Response, CBR: Control Benefit Rate.

## Data Availability

Not applicable.
